# Power outage mediates the associations between major storms and hospital admission of chronic obstructive pulmonary disease

**DOI:** 10.1186/s12889-021-12006-x

**Published:** 2021-10-29

**Authors:** Yanji Qu, Wangjian Zhang, Bo Ye, Samantha Penta, Guanghui Dong, Xiaoqing Liu, Shao Lin

**Affiliations:** 1 Guangdong Cardiovascular Institute, WHO Collaborating Center for Research and Training in Cardiovascular Diseases, Guangdong Provincial People’s Hospital, Guangdong Academy of Medical Sciences, Guangzhou, Guangdong China; 2 Department of Environmental Health Sciences, University at Albany, State University of New York, New York, NY USA; 3 Department of Epidemiology and Biostatistics, University at Albany, State University of New York, New York, NY USA; 4 College of Emergency Preparedness, Homeland Security and Cybersecurity, University at Albany, State University of New York, New York, NY USA; 5 Guangzhou Key Laboratory of Environmental Pollution and Health Risk Assessment, Guangdong Provincial Engineering Technology Research Center of Environmental and Health Risk Assessment, Department of Occupational and Environmental Health, School of Public Health, Sun Yat-sen University, Guangzhou, Guangdong China

**Keywords:** COPD, Mediation effect, Power outage, Storms

## Abstract

**Background:**

Chronic obstructive pulmonary disease (COPD) is the third-leading cause of death worldwide with continuous rise. Limited studies indicate that COPD was associated with major storms and related power outages (PO). However, significant gaps remain in understanding what PO’s role is on the pathway of major storms-COPD. This study aimed to examine how PO mediates the major storms-COPD associations.

**Methods:**

In this time-series study, we extracted all hospital admissions with COPD as the principal diagnosis in New York, 2001–2013. Using distributed lag nonlinear models, the hospitalization rate during major storms and PO was compared to non-major storms and non-PO periods to determine the risk ratios (RRs) for COPD at each of 0–6 lag days respectively after controlling for time-varying confounders and concentration of fine particulate matter (PM_2.5_). We then used Granger mediation analysis for time series to assess the mediation effect of PO on the major storms-COPD associations.

**Results:**

The RRs of COPD hospitalization following major storms, which mainly included flooding, thunder, hurricane, snow, ice, and wind, were 1.23 to 1.49 across lag 0–6 days. The risk was strongest at lag3 and lasted significantly for 4 days. Compared with non-outage periods, the PO period was associated with 1.23 to 1.61 higher risk of COPD admissions across lag 0–6 days. The risk lasted significantly for 2 days and was strongest at lag2. Snow, hurricane and wind were the top three contributors of PO among the major storms. PO mediated as much as 49.6 to 65.0% of the major storms-COPD associations.

**Conclusions:**

Both major storms and PO were associated with increased hospital admission of COPD. PO mediated almost half of the major storms-COPD hospitalization associations. Preparation of surrogate electric system before major storms is essential to reduce major storms-COPD hospitalization.

**Supplementary Information:**

The online version contains supplementary material available at 10.1186/s12889-021-12006-x.

## Background

Chronic obstructive pulmonary disease (COPD) is a progressive, incurable chronic lung disease and affects about 328 million people worldwide [1]. COPD exacerbation is an important cause of morbidity, impairment of health status, and mortality [2]. In the U.S., approximately 6.3% of adults had COPD, which affected 12–24 million Americans, resulting in about 120,000 deaths a year [3]. COPD is now the third-leading cause of death [4]. Deaths from chronic lung diseases increased by 50% from 1980 to 2010 in the US [5]. In addition, COPD is a typical medical condition which is sensitive to the environmental stressors, such as major storms, and symptoms of COPD are exacerbated during and after major storms and hospital admission of COPD increase accordingly [6–9]. However, potential pathways between major storms and increased COPD admission are unclear.

Extreme weather is projected to increase as a result of climate change, which is a growing global concern [9]. Major storms which mainly included flooding, thunder, hurricane, snow, ice, and wind were the most common extreme weather events and brought costly economic and health impacts worldwide [3, 4]. It was estimated that hurricanes, heat waves/droughts, tornadoes, and flooding were respectively associated with $392, $78, $46, and $30 billion losses in the U.S. from 2004 to 2013 [9]. Health impacts associated with these major storms included death, injury, adverse effects on mental health, and exacerbation of selected underlying medical conditions.

Because of their significant impact on critical infrastructure, major storms are considered one of the main causes of wide-area electrical disturbances worldwide and cause the majority of power outages (PO) [10]. Previous environmental health studies suggested that PO was associated with increased all-cause mortality and hospital admission of respiratory disease, especially COPD in the affected population [11–14]. A recent study found that PO was associated with a significantly elevated rate of COPD hospitalization, as well as greater costs and number of comorbidities of COPD [15].

Based on previous evidence, we anticipated that PO served as a mediator on the pathways between major storms and increased hospital admission of COPD (Supplementary Fig. 1). Estimating the potential mediation effect of PO would help policy makers and clinical practitioners to prepare surrogate electricity before major storms and allocate resources during weather events and prevent increased hospital admission of COPD accordingly. There are still several knowledge gaps to be addressed for the relationship among major storms, PO and COPD hospitalization. First, only a limited number of previous studies assessed the major storms-COPD and PO-COPD associations. Second, the distribution of major storms-induced PO was unavailable. Finally, the potential mediation effect of PO on the association between major storms and COPD has not been previously assessed. To fill these gaps, we conducted a time-series study in New York State to evaluate the direct and indirect effect of major storms on COPD and assess the mediation effect of PO on the major storms-COPD associations.

## Methods

### Study population and design

This study covered the entire population of New York State. All hospital admission records with a principal diagnosis of COPD between 2001 and 2013 were included. We focused on COPD because it was sensitive to environmental stressors and PO and the associations were biologically plausible. COPD patients are more sensitive than the general population to major storms [16]. Meanwhile, COPD patients usually rely on long-term oxygen therapy, and disconnection of oxygen support as a result of PO may exacerbate COPD symptom [15].

We used a time-series study to estimate the impact of major storms and PO on COPD, respectively. The impact was estimated by comparing the hospital admission of COPD during the event period to the non-event periods. With the time-series analysis, we were able to control important time-varying confounders and capture the cumulative health effect of major storms and PO on COPD, respectively [17, 18].

### Outcomes and data sources

We retrieved the hospital admission data of COPD between 1/1/2001–12/31/2013 from the New York Statewide Planning and Research Cooperative System (SPARCS). SPARCS is an on-going, legislatively mandated database covering 95% of hospitals across New York State [19, 20]. We retained records with a principal diagnosis of COPD (ICD-9 code: 491, 492, and 496) in the current study. We geocoded each selected hospitalization record based on the residential address reported to the SPARCS and assigned it to one of the 1742 operating divisions across the state, as defined by the Department of Public Service (DPS, http://www.dps.ny.gov/) according to the geographic coverage of different electricity providers. For each PO operating division, we obtained the total number of customers, the date, duration and cause of the PO that occurred, as well as the number of customers affected by the PO.

Major storms and other environmental variables, including daily temperature and humidity at the division level, were obtained from the Integrated Surface Database of the U.S. National Oceanic and Atmospheric Administration (NOAA, https://www.ncdc.noaa.gov/isd). We retrieved the daily ambient concentration of fine particulate matter (PM_2.5_) in each division from the Community Multiscale Air Quality Modeling System from the U.S. Environmental Protection Agency (https://www.epa.gov/cmaq) [21]. We linked all these datasets using a unique ID number for each DPS.

### Exposures and mediator

We defined major storms as the exposure. To access an adequate sample size to assess the association between major storms and hospital admission of COPD, we included and combined extreme weather events of flooding, thunderstorm, hurricane, snow, ice, and wind as the major storms. Scholars previously noted public health consequences of meteorological events like, blizzards, hurricanes, flooding, and thunderstorms [3, 4]. These types of events were also included in the list of events the NOAA’s National Weather Service used to develop its Storm Events Database (https://www.ncdc.noaa.gov/stormevents/pd01016005curr.pdf) [22].

PO was the mediator of interest in this study. We divided the number of customers affected by PO over the total number of customers to calculate the coverage of PO for each day in each division. We pooled all non-zero coverages across days and divisions to identify the 50th percentile. For the time-series analysis assessing the associations between PO and COPD in each division, we defined days with a coverage exceeding the 50th percentile as the exposure days, otherwise as control days.

### Statistical analysis

We first assessed the major storms-COPD and PO-COPD associations by fitting two Poisson models to estimate the excess increase of COPD admission due to major storms and PO in each electric power operation division, respectively. Specifically, we used the distributed lag nonlinear models with quasi-Poisson distribution in the time-series analysis [17, 18]. For each division, we regressed the daily number of cases against the indicator of exposure/control days, meanwhile adjusting for time-varying confounders including day of the week, holidays, the long-term trend and seasonality and weather confounders including temperature and humidity. We also controlled for the ambient concentration of PM_2.5_, the most important air pollutant in the model [23, 24]. The model used in this research was.

$$ Log\;(counts)= intercept+ cb\;(predictor)+ ns\ (time)+ holiday+ DOW+ ns\ \left( weather\ confounders\right)+ ns\ (PM2.5) $$

Counts was the daily hospital admissions of COPD, assuming a quasi-Poisson distribution; cb (predictor) was the cross-basis for the space and the lag dimension of major storms or PO; ns (time) was a natural spline fit to the days of the year; holiday and DOW were dummy variables representing whether a given day was a holiday and day of week, respectively; ns (weather confounders) was short for two natural spline fits to temperature and humidity, all with 3 degrees of freedom (df); ns (PM2.5) referred to a natural spline fit to the daily ambient concentration of particulate matter, 2.5 with 3 df. We estimated the effect (risk ratios, *RRs*) of major storms and PO on COPD within the first week (lag 0–6 days) following the events in each operating division, respectively. Longer lag time was not considered because we focused on assessing the acute or immediate effect of major storms and PO on the exacerbation of COPD symptoms based on biological mechanisms and prior literature. In addition, our data showed that the effects of both major storms and PO on COPD hospitalization peaked on lag day 2–3 and vanished after lag 6 day. We then pooled the estimates from each division using meta-analysis to generate the overall associations of major storms and PO, respectively, with COPD.

We then calculated the frequencies of PO according to different causes. We paid attention to the proportions of major storms among the all above-ground causes of PO. We also analyzed the most frequent contributors for PO among the major storms by calculating the frequencies of specific major storms induced PO.

We further performed single level Granger mediation analysis for time series to assess the causal mediation effect of PO on the association between major storms and COPD [25]. We performed granger mediation analysis for time series using the “gma” package in R [25]. The direct effect and indirect effect between major storms and COPD hospital admission were calculated in each division to estimate the percentage mediated by PO as the percentage mediated by PO = indirect effect / (direct effect + indirect effect). We then pooled these estimators across all the operating divisions using the meta-analysis to generate the overall mediation effect of PO on the major storms-COPD associations. We conducted meta-analyses using “rma” function of “metafor” package in R [26]. We completed geocoding using the Street and Address Maintenance Program in ArcGIS 10.3.1 [27] and accomplished all the analyses using R 3.4.1.

This study was approved by the Institutional Review Board at the University at Albany, State University of New York (approval number 17X189).

## Results

**Fig. 1:**
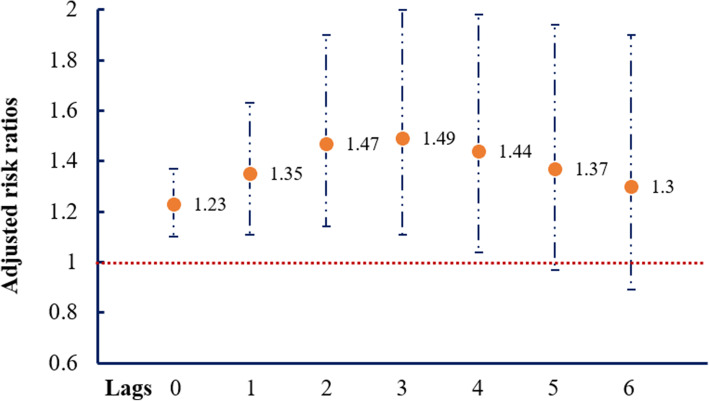
Associations (adjusted risk ratios, RRs) ^†^ between major storms with hospital admission due to chronic obstructive pulmonary disease, NY, 2001–2013**.** ^†^ Adjust for the cross-basis for the exposure period [cb (period)], splines with 5 df per year [ns (time)], splines with 3 df of temperature [ns (temp)] and dew point [ns (dewp)], indicators for holidays and day of the week, and ambient concentration of PM_2.5_

**Fig. 2:**
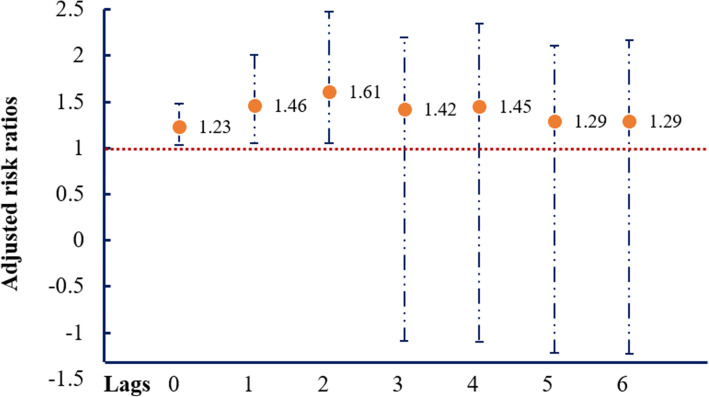
Associations (adjusted risk ratios, RRs) ^†^ between power outage with hospital admission due to chronic obstructive pulmonary disease, NY, 2001–2013**.** ^†^ Adjust for the cross-basis for the exposure period [cb (period)], splines with 5 df per year [ns (time)], splines with 3 df of temperature [ns (temp)] and dew point [ns (dewp)], indicators for holidays and day of the week, and ambient concentration of PM_2.5_

**Fig. 3:**
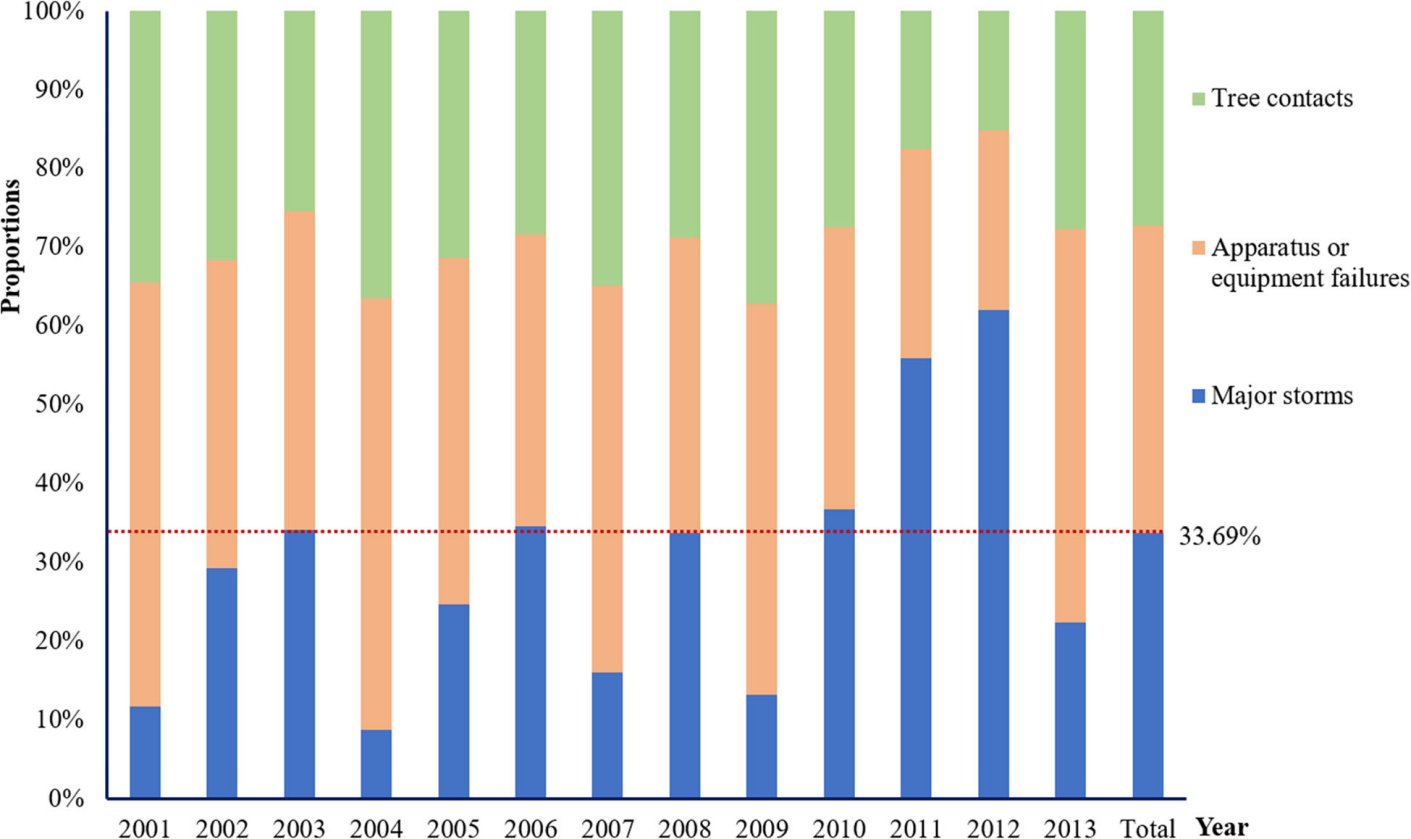
Above ground causes of total power outage and the proportion of major storms among the causes, NY, 2001–2013

**Fig. 4:**
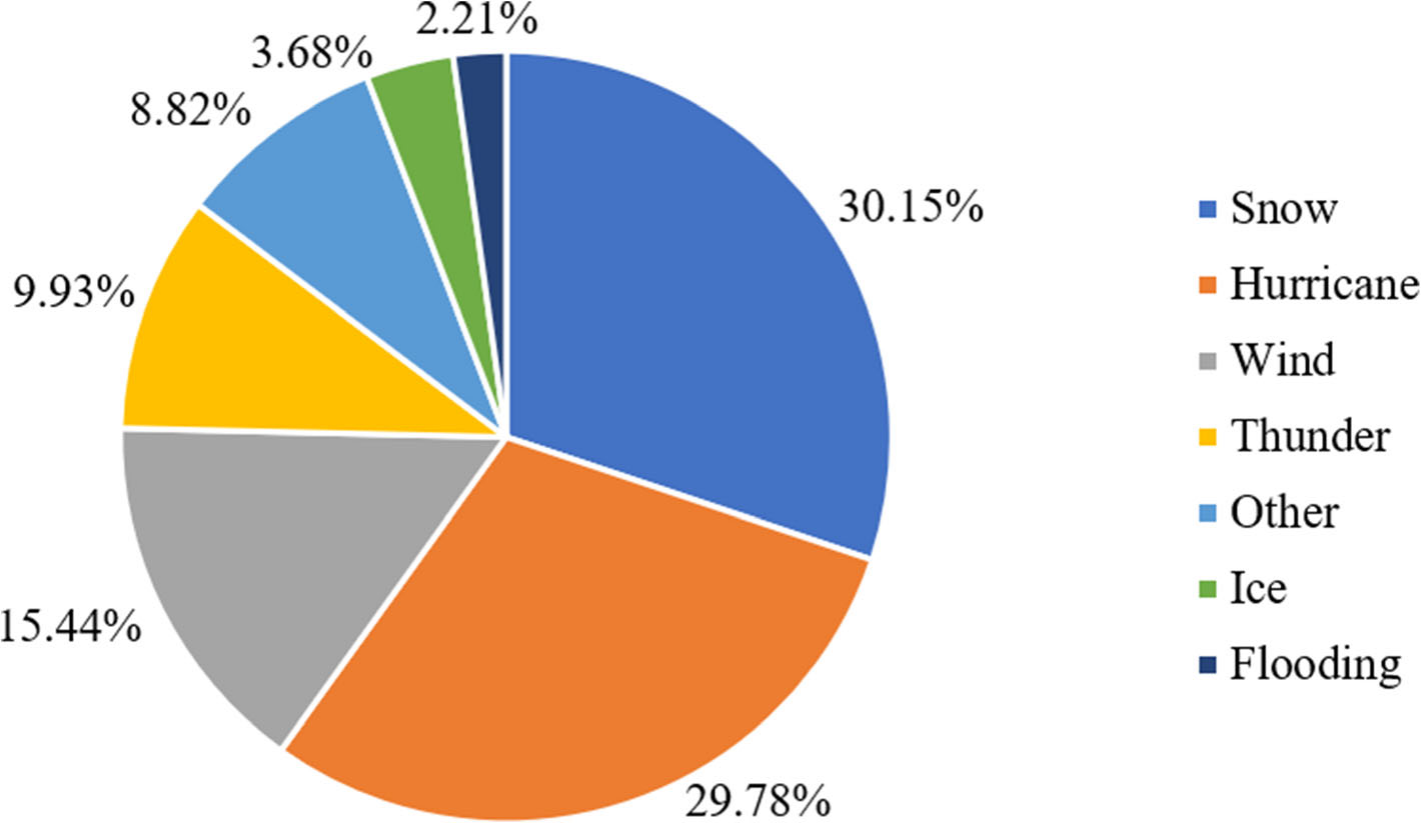
Compositions of major storms as the causes of power outage, NY, 2001–2013

The relationships among major storms (exposure), PO (mediator) and COPD hospital admission (outcome) are illustrated in Supplemental Fig. 1. We first presented the associations of major storms-COPD (Fig. 1), PO-COPD (Fig. 2) and major storms-PO (Figs. 3 and 4) in the following results, respectively. Then, by considering all these associations, mediation analysis was conducted, and the results are presented in Table 1.

**Table 1:** Mediation effect of power outage on the associations between major storms and chronic obstructive pulmonary disease, NY, 2001–2013

Lags	Direct effect ^a^	Indirect effect ^b^	Estimated percent mediated by PO (%, 95CI)
0	0.21 (0.13,0.29)	0.39 (0.27,0.50)	65.0 (48.7,79.3)
1	0.15 (0.08,0.23)	0.19 (0.13,0.25)	55.4 (36.7,76.2)
2	0.13 (0.05,0.21)	0.13 (0.08,0.17)	49.6 (28.5,76.5)
3	0.15 (0.07,0.22)	0.17 (0.11,0.23)	53.4 (31.9,77.0)
4	0.16 (0.08,0.23)	0.21 (0.14,0.28)	57.7 (37.9,78.2)
5	0.17 (0.09,0.25)	0.29 (0.19,0.39)	62.7 (42.1,81.6)
6	0.15 (0.06,0.24)	0.21 (0.13,0.28)	57.8 (36.1,81.4)

^a^ Direct effect: ADE, Average Direct Effects;

^b^ Indirect effect: ACME, Average Causal Mediation Effects

As shown in Fig. 1, the RRs of COPD administration following major storms ranged from 1.23 to 1.49. The significant effect of major storms on COPD lasted for 4 days. The risk was strongest at lag3 (RR = 1.49, 95% CI: 1.11–2.00), followed by lag2 (RR = 1.47, 95% CI: 1.14–1.90), lag4 (RR = 1.44, 95% CI: 1.04–1.98), lag1 (RR = 1.35, 95% CI: 1.11–1.63) and lag0 (RR = 1.23, 95% CI: 1.10–1.37).

The associations between PO and hospital admission of COPD were presented in Fig. 2. We observed a significantly increased hospital admission of COPD in the first 3 days after PO (RRs ranged from 1.23 to 1.61). The RRs of COPD was the strongest on lag2 day after PO (RRs = 1.61, 95% CI: 1.05–2.48), followed by lag1 (RRs = 1.46, 95% CI: 1.05–2.01) and lag0 (RRs = 1.23, 95% CI: 1.03–1.48).

As shown in Fig. 3, major storms were important causes of PO, responsible for 33.69% of the all above-ground causes of PO events. Among the major storms, snowstorm contributed the most of PO with a percentage of 30.15%, followed by hurricane (29.78%), wind (15.44%), thunder (9.93%), others (8.82%), ice (3.68%), and flooding (2.21%), respectively (Fig. 4).

The direct effect, indirect effect and estimated percent mediated by PO of the associations between major storms and COPD hospitalization was shown in Table 1. We found stronger indirect effects (from 0.13 on lag2 day to 0.21 on lag0 day) of major storms on COPD than direct effects (from 0.13 on lag2 day to 0.39 on lag0 day), consistently from lag0 to lag6. Thus, we found significant mediation effect of PO on the major storms-COPD associations on lag0–6 days (percent mediated by PO ranged from 49.6% on lag2 day to 65% on lag0 day, *P* < 0.001).

## Discussion

### Major storms and COPD

We found that major storms were associated with 1.23 to 1.49 higher hospital admission of COPD from the lag0 to lag4 day after the major storms. The effects of major storms on COPD increased from lag0 continuously to lag3 and lasted to lag4. Among the major storms, snow, hurricane, wind and thunderstorms were the top causes of PO in New York State.

Similar to our findings, previous studies also indicated that COPD patients were vulnerable to the major storms [6–9]. For COPD patients with impaired lung function whose mobility is limited by fatigue or the use of supplemental oxygen tanks, even a small flood could be a debilitating event. Worsening COPD problems were reported as the aftermath of Hurricane Katrina which was a very large and powerful Category 3 storm that hit the Gulf Coast region in 2005 [6]. The other deadliest storm, hurricane Sandy, which particularly impacted New Jersey and the greater New York City area in 2012, was associated with 1.28–1.45 higher emergency department admission of COPD in varied primary payer classes [28]. In addition, highly windy day and thunderstorms were also potential triggers of COPD symptoms. Thunderstorms are known to trigger asthma symptoms, implying the possibility to flare COPD symptoms [29]. Such effects are stronger when storms arrive during springtime’s high-pollen periods. The pollen grains get sucked into storm clouds and become saturated with water. They then break into smaller grains that are carried by wind at the ground level and easily inhaled into the lungs. In addition, other environmental factors, such as extremes of temperature, both heat and cold, were associated with increased respiratory morbidity and mortality in COPD [30, 31]. Previous studies suggested that 18 °C (64 °F) might be a threshold below which colder temperatures adversely impact respiratory health for COPD patients [32, 33]. During winter storms (snow and ice), the temperature is much lower than this threshold, especially when heater and air conditioning systems are cannot function during major storms-induced PO. It was also indicated that each degree temperature above the threshold of the temperature-health effect curve (29 °C–36 °C) was associated with a 2.7–3.1% increase in same-day hospitalizations due to respiratory disease, including COPD [34].

### PO and COPD

We found that PO was associated with 1.23 to 1.61 higher hospital admission of COPD from lag0 to lag2 day after the event. The association between PO and COPD was the strongest at lag2 day, followed by lag1 and lag0 day.

Our results were similar to the previous studies which indicated that storms-related PO were associated with increased rates of hospitalization (incidence rate ratio ranged from 2.41 to 8.17 for different COPD phenotypes comparing blackout days and normal days) [14]. A prior study found that the 2003 Northeast Blackout was associated with the most striking increases of respiratory hospital admissions among COPD patients [14]. A recent study determined the impact of PO on COPD exacerbations and found that PO was associated with 3–39% increase of COPD hospitalization (RR ranged from 1.03 to 1.39) [15]. This study found that the risk of PO on COPD was strongest at lag0 and lag1 days, which was similar to our results. COPD patients, who were reliant on electrically powered medical equipment like ventilators and oxygen were considered to be especially vulnerable to the PO [13, 35, 36].

### Mediation effect of PO

We found that about 1/3 PO were induced by major storms and approximately half of the associations between major storms and hospital admission of COPD could be explained by the pathway of PO. The indirect effect of major storms was stronger than the direct effect on COPD. No previous studies assessed the mediation effect of PO on the major storms-COPD hospital admission associations. Thus, comparison to prior research may be impossible.

There are several potential mechanisms through which PO mediates the association between major storms and hospital admission of COPD. First, individuals with COPD may be dependent on at-home oxygen concentrators which require electricity to work. If power is knocked out by a storm, that could aggravate symptoms of COPD, with hospital admissions of COPD increasing accordingly. Second, COPD symptoms may be exacerbated because of inhaling dust and debris in the air during and after the storms. Among individuals with COPD, outdoor air pollutants, i.e., particulate matter, were associated with loss of lung function, increased respiratory symptoms, and mortality [37]. When storms are accompanied by PO, indoor and outdoor air quality further deteriorates from the use of backup electric generators, many of which burn diesel fuel to make electricity. Diesel Burning creates air pollutants, like particulate matter and nitrogen oxides, which make air quality worse after major storms. The nitrogen oxides that generators emit combine with other compounds in the presence of sunlight and generate ozone, which is a potent trigger for COPD attacks [37]. These irritants all trigger symptom flare-ups and increase the hospital admission of COPD. Third, electricity is essential to multiple systems, and a failure in the electrical grid brings cascading effects on water and sewage treatment, transportation, and especially health care systems [36, 38]. Many medical offices in hard hit areas closed after hurricane Katrina, leaving residents without access to prescribed essential medications for chronic breathing problems [33]. Surveys showed that COPD patients were one of the groups that were particularly affected by Hurricane Katrina [6]. Treatment interruptions and lack of access to medication for COPD patients could exacerbate respiratory symptoms both during and after the extreme event [35]. Fourth, the deteriorating residence environment as a consequence of major storms can get worse during PO which may induce COPD exacerbation. For example, bacteria and virus infection, which is the leading cause of overall COPD exacerbation, increases after major storms because mold begins to bloom on wet walls and floors [39]. PO makes this problem more serious because electricity dependent air conditioning systems and dehumidifiers stop working. In addition, during major storms-associated PO, COPD patients may not only be exposed to more allergens, but may also be required to endure extreme hot or cold temperatures which themselves have considerable effects on COPD [40]. A meta-analysis found that the hospitalization of COPD was 5% higher for every 5 °C increase in daily mean temperature. Previous study of a big data analysis suggested that cold weather increased viral infection and air pollution and was also a significant risk factor for COPD exacerbation [41].

### Implications for practice

These findings emphasize the public health importance of maintaining electric power during and after major storms, and in preparing electrical systems to withstand extreme wind, temperature, and precipitation associated health events. They offer important guidance for practitioners of emergency public health and those who serve COPD patients in designing and implementing practices and allocating resources to prepare for major storms-induced PO events, and by extension, to reduce COPD admissions. In addition, at the household level, families with COPD patients are strongly encouraged to prepare surrogate electricity (e.g., generators and/or back-up battery power for medical equipment) or plan suitable temporary residence and transportation mode before major storms and PO event.

### Strength and limitations

We are the first to assess the mediation effect of PO on the association between major storms and hospital admission of COPD. Both exposures and outcomes were extracted from existing objective data and reporting bias was minimized. We captured a large group of COPD patients from a legislatively mandated database covering 95% of hospitals across New York State during more than 10 years. We accessed multiple environmental information from different sources and integrated them in the assessment.

Although our study provides new insights, several limitations should be considered when interpreting our results. First, we only included hospital admissions (no outpatient visits or emergency department visits) of COPD, which merely captured the tip of iceberg. However, as exacerbation of COPD symptoms is generally severe and requires immediate medical care and treatment through hospital admission, our study population including COPD hospitalization may be appropriate in capturing the most severe group. Second, air pollution during major storms and PO may be confounders in the storms/PO-COPD associations [42]. Nevertheless, we have minimized the confounding effect by controlling for ambient concentration of PM_2.5_ at the county level in the statistical analysis. Third, the models we used to estimate the overall effect of major storms and PO on COPD may not be confirmed by typical model diagnostics. We obtained the overall effect of major storms and PO on COPD by developing a model for each of the specific divisions where the major storms and PO occurred and then pooling the results from these different divisions through meta-analysis. We followed the standard method widely used in previous studies with similar design [15, 43, 44]. Fourth, we only examined the acute or immediate effect (RRs) within the first week (lag 0–6 days) following the events. However, we observed that the effects of both major storms and PO on COPD hospitalization vanished after lag 6 day. In addition, we focused on assessing the immediate effect of major storms and PO on the exacerbation of COPD symptoms by including hospital admissions of COPD which represent the most severe conditions and need immediate medical care. Longer lag time may be considered in future studies. Fifth, we did not conduct sensitivity analysis by parameters in defining our model, including knots and degree of freedom. However, the logknots function that we used is a standard methodology procedure and the most used function to determine the location of knots in the lag space [45]. Meanwhile, we used df = 3 in our analysis and conducted extensive sensitivity analysis in the same database previously and found very similar effect estimates [15]. Finally, this is an observational and ecological study in nature, residual confounding in individual level may exist and temporal consequence cannot be determined. Future studies in individual level with better study design are needed to confirm our findings.

## Conclusions

Both major storms and PO were associated with increased hospital admission of COPD. Major storms were one of the most common causes of PO. PO mediated almost half of the associations between major storms and COPD hospital admission. Preparation of surrogate electric system before major storms are essentially important to alleviate increase of hospital admission due to COPD.

## Supplementary Information

**Additional file 1.**

## Data Availability

The data used during the current study are available from the corresponding author (slin@albany.edu) on reasonable request.

## Abbreviations

COPD: chronic obstructive pulmonary disease
ICD-9: International Classification of Diseases, Ninth Revision
PO: power outages
RR: rate ratio
SPARCS: the New York Statewide Planning and Research Cooperative System

## References

[1] Quaderi SA, Hurst JR (2018). The unmet global burden of COPD. Glob Health Epidemiol Genom.

[2] Qureshi H, Sharafkhaneh A, Hanania NA (2014). Chronic obstructive pulmonary disease exacerbations: latest evidence and clinical implications. Ther Adv Chronic Dis.

[3] Landesman LY, Burke RV. Landesman’s Public Health Management of Disasters: The Practice Guide. 4th ed. Washington, DC: American Public Health Association Press; 2017.

[4] McKinney S, Papke ME. Public health emergency preparedness: a practical approach for the real world. Burlington: Jones & Bartlett Learning; 2019.

[5] Centers for Disease Control and Prevention (CDC). Chronic obstructive pulmonary disease among adults--United States. MMWR Morb Mortal Wkly Rep. 2011:2012. 23169314

[6] Arrieta MI, Foreman RD, Crook ED, Icenogle ML (2009). Providing continuity of care for chronic diseases in the aftermath of Katrina: from field experience to policy recommendations. Disaster Med Public Health Prep.

[7] Platz E, Cooper HP, Silvestri S, Siebert CF (2007). The impact of a series of hurricanes on the visits to two Central Florida emergency departments. J Emerg Med.

[8] Kobayashi S, Fujimoto K (2018). Exacerbation of COPD by air pollution, cold temperatures, or discontinuation of medicine: what should be measured to help prevent it. Disaster and respiratory diseases. Respiratory disease series: diagnostic tools and disease manage.

[9] Bell JE, Herring SC, Jantarasami L, Adrianopoli C, Benedict K, Conlon K (2016). Ch. 4: impacts of extreme events on human health. The impacts of climate change on human health in the United States: a scientific assessment. III.

[10] Curtis S, Fair A, Wistow J, Val DV, Oven K (2017). Impact of extreme weather events and climate change for health and social care systems. Environ Health.

[11] Kishore N, Marqués D, Mahmud A, Kiang MV, Rodriguez I, Fuller A, et al. Mortality in Puerto Rico after hurricane Maria. N Engl J Med. 2018;379(2):162–70. 10.1056/NEJMsa1803972. 10.1056/NEJMsa1803972 29809109

[12] Christine D, Kathryn L, Sarah J, Kazuhiko I, Thomas M (2019). Health impacts of citywide and localized power outages in new York City. Environ Health Perspect.

[13] Anderson GB, Bell ML (2012). Lights out: impact of the august 2003 power outage on mortality in New York. NY Epidemiology.

[14] Lin S, Fletcher BA, Luo M, Chinery R, Hwang S-A (2011). Health impact in new York City during the northeastern blackout of 2003. Public Health Rep.

[15] Zhang W, Sheridan SC, Birkhead GS, Croft DP, Brotzge JA, Justino JG, et al. Power outage: an ignored risk factor for COPD exacerbations. Chest. 2020;158:2346–57. 10.1016/j.chest.2020.05.555 PMC7768937 32502591

[16] Crimmins AJ, Balbus JL, Gamble DR, Easterling KL, Ebi J, Hess KE, et al. Appendix 1: Technical Support Document: Modeling Future Climate Impacts on Human Health. The Impacts of Climate Change on Human Health in the United States: A Scientific Assessment. Washington, DC: U.S. Global Change Research Program; 2016. p. 287–300.

[17] Gasparrini A, Armstrong B, Kenward MG (2010). Distributed lag non-linear models. Stat Med.

[18] Bhaskaran K, Gasparrini A, Hajat S, Smeeth L, Armstrong B (2013). Time series regression studies in environmental epidemiology. Int J Epidemiol.

[19] Rich DQ, Zhang W, Lin S, Squizzato S, Thurston SW, van Wijngaarden E, et al. Triggering of cardiovascular hospital admissions by source specific fine particle concentrations in urban centers of New York state. Environ Int. 2019;126:387–94. 10.1016/j.envint.2019.02.018. 10.1016/j.envint.2019.02.018 PMC6441620 30826617

[20] Zhang W, Lin S, Hopke PK, Thurston SW, van Wijngaarden E, Croft D, et al. Triggering of cardiovascular hospital admissions by fine particle concentrations in New York state: before, during, and after implementation of multiple environmental policies and a recession. Environ Pollut. 2018;242(Pt B):1404–16. 10.1016/j.envpol.2018.08.030. 10.1016/j.envpol.2018.08.030 30142556

[21] Lobdell DT, Jagai JS, Rappazzo K, Messer LC (2011). Data sources for an environmental quality index: availability, quality, and utility. Am J Public Health.

[22] National Oceanic & Atmospheric Administration, National Weather Service. 2016. National Weather Service Instruction 10–1605, operations and services performance, NWSPD 10–16. Storm data preparation. Retrieved March 3, 2021 at https://www.ncdc.noaa.gov/stormevents/pd01016005curr.pdf.

[23] Zhang W, Du Z, Zhang D, Yu S, Huang Y, Hao Y. Assessing the impact of humidex on HFMD in Guangdong Province and its variability across social-economic status and age groups. Sci Rep. 2016;6(1):18965. 10.1038/srep18965. 10.1038/srep18965 PMC4705518 26743684

[24] Xiao X, Gasparrini A, Huang J, Liao Q, Liu F, Yin F, et al. The exposure-response relationship between temperature and childhood hand, foot and mouth disease: a multicity study from mainland China. Environ Int. 2017;100:102–9. 10.1016/j.envint.2016.11.021. 10.1016/j.envint.2016.11.021 28069250

[25] Zhao Y, Luo X (2017). Granger mediation analysis of multiple time series with an application to fMRI.

[26] Viechtbauer W. Conducting meta-analyses in R with the metafor. J Stat Softw. 2010;36(3). 10.18637/jss.v036.i03.

[27] The NYS GIS Program office (2017). The Street and Address Maintenance (SAM) Program.

[28] Mongin SJ, Baron SL, Schwartz RM, Liu B, Taioli E, Kim H (2017). Measuring the impact of disasters using publicly available data: application to hurricane Sandy (2012). Am J Epidemiol.

[29] Al-Rubaish AM (2007). Thunderstorm-associated bronchial asthma: a forgotten but very present epidemic. J Family Commun Med.

[30] Schwartz J (2005). Who is sensitive to extremes of temperature?: a case-only analysis. Epidemiology.

[31] McCormack MC, Paulin LM, Gummerson CE, Peng RD, Diette GB, Hansel NN (2017). Colder temperature is associated with increased COPD morbidity. Eur Respir J.

[32] Jevons R, Carmichael C, Crossley A, Bone *A minimum* indoor temperature threshold recommendations for English homes in winter – A systematic review. 2016;136:4–12. 10.1016/j.puhe.2016.02.007. 10.1016/j.puhe.2016.02.007 27106281

[33] Kessler RC, Group HKCA (2007). Hurricane Katrina’s impact on the care of survivors with chronic medical conditions. J Gen Intern Med.

[34] Lin S, Luo M, Walker RJ, Liu X, Hwang S-A, Chinery R (2009). Extreme high temperatures and hospital admissions for respiratory and cardiovascular diseases. Epidemiology.

[35] Lane K, Charles-Guzman K, Wheeler K, Abid Z, Graber N, Matte T (2013). Health effects of coastal storms and flooding in urban areas: a review and vulnerability assessment. J Environ Public Health.

[36] Klinger C, Landeg O, Murray V. Power outages, extreme events and health: a systematic review of the literature from 2011-2012. PLoS Curr. 2014;6. 10.1371/currents.dis.04eb1dc5e73dd1377e05a10e9edde673. 10.1371/currents.dis.04eb1dc5e73dd1377e05a10e9edde673 PMC3879211 24459613

[37] Sunyer J (2001). Urban air pollution and chronic obstructive pulmonary disease: a review. Eur Respir J.

[38] Cutter SL, Solecki W, Bragado N, Carmin J, Fragkias M, Ruth M, et al. Ch. 11: Urban systems, infrastructure, and vulnerability. Climate Change Impacts in the United States: The Third National Climate Assessment. Washington, DC: U.S. Global Change Research Program; 2014.

[39] Wedzicha JA, Seemungal TAR. COPD exacerbations: defining their cause and prevention. Lancet. 2007;370:786–96. 10.1016/S0140-6736(07)61382-8 PMC7134993 17765528

[40] Hansel NN, McCormack MC, Kim V. The Effects of Air Pollution and Temperature on COPD. COPD. 2016;13(3):372–9. 10.3109/15412555.2015.1089846 PMC4878829 26683097

[41] Lee J, Jung HM, Kim SK, Yoo KH, Jung KS, Lee SH, et al. Factors associated with chronic obstructive pulmonary disease exacerbation, based on big data analysis. Sci Rep. 2019;9(1):6679. 10.1038/s41598-019-43167-w PMC6491439 31040338

[42] Marufu LT, Taubman BF, Bloomer B, Piety CA, Doddridge BG, Stehr JW, et al. The North American electrical blackout: an accidental experiment in atmospheric chemistry. Geophys Res Lett. 2003;2004(13). 10.1029/2004GL019771.

[43] Guo Y, Gasparrini A, Armstrong BG, Tawatsupa B, Tobias A, Lavigne E, et al. Temperature variability and mortality: a multi-country study. Environ Health Perspect. 2016;124(10):1554–9. 10.1289/EHP149. 10.1289/EHP149 PMC5047764 27258598

[44] Gasparrini A, Guo Y, Hashizume M, Lavigne E, Zanobetti A, Schwartz J, et al. Mortality risk attributable to high and low ambient temperature: a multicountry observational study. Lancet. 2015;386(9991):369–75. 10.1016/S0140-6736(14)62114-0. 10.1016/S0140-6736(14)62114-0 PMC4521077 26003380

[45] Gasparrini A (2011). Distributed lag linear and non-linear models in R: the package dlnm. J Stat Softw.

