# Supplementary material for: Power outage mediates the associations between major storms and hospital admission of chronic obstructive pulmonary disease
Source: BMC Public Health. 2021 Oct 29;21:1961. doi: 10.1186/s12889-021-12006-x (PMC8556928; doi:10.1186/s12889-021-12006-x)
Supplement: Supplementary file 1 — Additional file 1. [file 12889_2021_12006_MOESM1_ESM.docx]

Indirect effect

Major storms

COPD admission

Major storms

Power outage

COPD admission

Total effect

Direct effect

Proportion mediated= Indirect effect/Total effect

**e-Figure 1.** Illustration of power outage as a mediator in the association between major storms and hospital admission due to chronic obstructive pulmonary disease, NY, 2001-2013
